# German Index of Socioeconomic Deprivation (GISD): Revision, update and applications

**DOI:** 10.25646/10641

**Published:** 2022-12-09

**Authors:** Niels Michalski, Marvin Reis, Fabian Tetzlaff, Meik Herber, Lars Eric Kroll, Claudia Hövener, Enno Nowossadeck, Jens Hoebel

**Affiliations:** 1 Robert Koch Institute, Berlin Department of Epidemiology and Health Monitoring; 2 Zentralinstitut für die kassenärztliche Versorgung in Deutschland, Fachbereich Data Science und Versorgungsanalysen

**Keywords:** SOCIAL DEPRIVATION, REGIONAL INEQUALITY, HEALTH INEQUALITY, GISD, INKAR

## Abstract

**Background:**

Regional deprivation indices enable researchers to analyse associations between socioeconomic disadvantages and health outcomes even if the health data of interest does not include information on the individuals’ socioeconomic position. This article introduces the recent revision of the German Index of Socioeconomic Deprivation (GISD) and presents associations with life expectancy as well as age-standardised cardiovascular mortality rates and cancer incidences as applications.

**Methods:**

The GISD measures the level of socioeconomic deprivation using administrative data of education, employment, and income situations at the district and municipality level from the INKAR database. The indicators are weighted via principal component analyses. The regional distribution is depicted cartographically, regional level associations with health outcomes are presented.

**Results:**

The principal component analysis indicates medium to high correlations of the indicators with the index subdimensions. Correlation analyses show that in districts with the lowest deprivation, the average life expectancy of men is approximately six years longer (up to three years longer for women) than for those from districts with the highest deprivation. A similar social gradient is observed for cardiovascular mortality and lung cancer incidence.

**Conclusions:**

The GISD provides a valuable tool to analyse socioeconomic inequalities in health conditions, diseases, and their determinants at the regional level.

## 1. Introduction

The spatial distributions of health chances, risks of disease and mortality rates are of integral importance for public health monitoring and social epidemiological research. Pronounced regional differences for various health indicators are documented for Germany [1–3]. Socioeconomic factors provide a major foundation for explaining these differences because health chances and risks of disease are closely associated with socioeconomic disadvantages on the individual as well as on the spatial level. The socially inequitable distribution of health chances, risks of disease, mortality, and life expectancy is a worldwide phenomenon. The World Health Organisation (WHO) defines the reduction of socioeconomic health-related inequalities as a central development goal for the improvement of health and quality of life for all people [4–6]. In Germany, individual socioeconomic disadvantage is also associated with poorer self-reported health, with more risky behaviour with regard to health, and with higher disease burden and mortality [7–9]. A large portion of the regional health-related differences can already be explained by the spatial distribution of socioeconomic factors, i.e. the differences between regions with regard to the socioeconomic status of their inhabitants [10]. In regions with more socioeconomically disadvantaged inhabitants, the morbidities are thus higher when these people have higher risks of disease because of their individual socioeconomic situation. Additionally, multilevel analyses have revealed independent effects of regional socioeconomic disadvantage on health chances and risks of disease, which go beyond the spatial aggregation of individual characteristics. These contextual effects originate, for example, from environmental factors of the residential area [9]. The literature specifically names living conditions at the place of residence, such as traffic volume, crime rates, recreational opportunities, or sports facilities, which often depend on economic and political conditions for their spatial distribution [9, 11–13].

To document the extent of regional health-related inequalities, and to identify regions with particular need for prevention and care, regional deprivation indices have been developed in many countries. The term regional deprivation is used here in accordance with the notion of social deprivation and identifies the level of disadvantage of the residential population in a region resulting from a relative lack of socioeconomic resources, from a comparatively high socio-spatial burden as well as from corresponding limited opportunities for social participation. The measurement of regional social deprivation originated in England in the 1980s where such indices were initially used to assess regional differences in health care needs [14, 15]. While early indices were limited to socioeconomic factors [15–17], more recent indices of multiple deprivation also included additional indicators concerning the people’s living conditions or regional crime rates [18].

In 2017, the German Index of Socioeconomic Deprivation (GISD) was developed by the Robert Koch-Institute (RKI), in order to illustrate regional socioeconomic inequalities in health and with the intention to spark the exploration of the causes of regional socioeconomic health differences [10]. Deprivation is thereby understood as a relative disadvantage attached to spatial units within Germany or within individual federal states. The GISD serves as a measure of the relative position of Germany’s administrative regions with regards to their populations’ socioeconomic situation. The indicators of the GISD were selected so that they permit analogy with the individual socioeconomic status, an important concept in social epidemiology, which comprises the dimensions education, occupation, and income [14, 19, 20]. The GISD allows to analyse socioeconomic differences in health chances, risks of disease and mortality rates in Germany even if the respective health data does not include any information relating to the individual socioeconomic status. Since its development, the index has been linked to various health data in order to perform studies on the association between socioeconomic and health characteristics at the spatial level with aggregated individual data. This proceeding has been applied to population-based cancer registry data in Germany [21, 22], official notification data on various infectious diseases [23–25], ambulatory claims data [26], and regional mortality and life expectancy data [10]. By linking the GISD, it became possible to tap into these data sources in order to perform social epidemiological analyses, which had previously been neglected due to a lack of socioeconomic information in the data. The GISD was also used in multilevel analyses in order to analyse associations of regional socioeconomic deprivation and health in addition to effects of the individual socioeconomic status. For example, data from the RKI health surveys and from the school entry health examinations were used for this purpose [10, 11, 27].

This article presents the first comprehensive revision of the GISD (version 2022 v1). It uses more current data, introduces an additional indicator, and optimises the data harmonisation. At first, the used indicators, the data basis, the aggregation rules, and the weighting of the indicators will be presented. As a next step, the regional distribution will be shown by means of maps, and correlation analyses will be introduced based on examples. Finally, the limitations of the index and perspectives of its use will be discussed.

## 2. Methods

### 2.1 Data basis

Data from the INKAR database (Indicators, Maps and Graphics on Spatial and Urban Monitoring) of the Federal Institute for Research on Building, Urban Affairs, and Spatial Development (BBSR) [28] is used to generate the GISD. INKAR is an interactive online atlas containing regional statistical information on the topics of population development, job market, education, economy, housing, traffic, and the environment.

Roughly 600 indicators for various regional levels are stored in the database, allowing for comparisons between European regions, federal states, districts, and municipalities. The breakdown of the regional units is based on the administrative division of Germany (Table 1). The statistics comprise time series data for the years 1995 and onwards. Due to its public availability and comprehensive documentation, the INKAR data offers a high level of transparency. The data is also harmonised over time and is based on the current territorial statuses, so that time trends can be analysed. Based on the mentioned characteristics, data from the INKAR database is preferred compared to other data sources. The data used for this article refers to the territorial status as of 31 December 2019 and includes values from 1998 to 2019.

Moreover, information relating to life expectancy at the level of districts and independent cities for the most current available time period (2016/2017) was taken from the INKAR database for the exemplary analyses of associations with the GISD. Data from the Centre for Cancer Registry Data (ZfKD) at the RKI was used to analyse socioeconomic inequalities in lung cancer incidence, and socioeconomic inequalities in cardiovascular mortality was examined on the basis of the German official Cause-of-death statistics (TUS). The cancer registry data as well as the TUS data was initially aggregated at the district level and then linked with the GISD. Age-standardised incidence and mortality rates were calculated stratified by GISD quintiles.

### 2.2 Indicators

The revised index essentially uses the indicators from the original version of the GISD [10]. The rationale for the selection was based on the results of a systematic literature search in the PubMed and Google Scholar databases [10]. The indicators should moreover meet three further criteria: Firstly, the regional resolution of the data should be as finegrained as possible. It should be available at least at the district level, ideally at the level of municipalities and collective municipalities (GVB). Secondly, the indicators should be available over a time period between the most recent year and annually up to 20 years backwards while being assigned to current territorial statuses. Thirdly, the indicators should correlate with their respective subdimensions with sufficient strength in order to justify a combination into the dimensions. For the update of the GISD, the indicators currently provided in the INKAR database were analysed with regard to their suitability. It turned out that all eight originally used indicators are still included in the INKAR database. The indicator ‘proportion of employees subject to social insurance contributions with university degree as share of all subject to social insurance contributions’ turned out to be problematic because this indicator was no longer provided based on the place of residence in the used database version of INKAR. Instead it was based on the place of work. The reference to the place of residence is without alternative for the indicator if the indicator is to be considered an approximate measure for the average level of education among the regional resident population. In consequence, for the current GISD revision, the indicator time series based on the place of residence were obtained directly from the Statistics of the Federal Employment Agency [29]. Following on from this, the indicator ‘proportion of employees subject to social insurance contributions without professional qualification out of all employees subject to social insurance contributions’ was also included in the list of indicators. In the present revision, the education dimension just as the income and employment dimension can henceforth be represented by three indicators. To account for the lack of analogy between the indicators of the employment dimension and those of the occupational dimension of socioeconomic status (SES), the labelling of this GISD dimension differs from that of SES. Table 2 illustrates the dimensions with their indicators and the respective original source of the data.

For three of the nine indicators, data at the level of municipalities and collective municipalities (GVB) are available, so that the index can determine differences in the socioeconomic deprivation between GVB within districts. Due to the fact that this small-scale variation is based on only one third of the index-forming indicators, the index for the level of the GVB remains associated with greater uncertainties than for the district level. For the indicators ‘unemployment rate’ and ‘employment rate’, the data for the years from 1998 to 2000 was only available at the district level, so that the variation between GVB within districts is lower for these early years. For the education indicators ‘employees without qualification’ and ‘employees with university degree’, no values for the year 2013 are provided due to a changeover in the reporting procedure on social insurance [30]. To fill this time gap, the data for 2011 was carried forward to 2012. For some indicators, the time series do not start in the base year 1998. Values were estimated on the basis of the available time series (linear random intercept models for time series) for these indicators. This relates to the years prior to 2004 and in particular to the years 1998 to 2000, for which three to five indicators were replaced accordingly. From 2001 to 2003, only missing values of one indicator had to be estimated.

To be able to use the information of the raw data as time series and to prepare it for the principal component analysis, some indicators were adjusted for artefacts of the survey and statistical artefacts: 1) The indicators with currency-based open-ended scales (tax revenue, gross wage, and average net household income) were adjusted to account for purchasing power and natural logarithms were taken. 2) For the indicators ‘employees without certificate’ and ‘employees with university degree’, the already mentioned changeover in the reporting procedure on social insurance [30] lead to an interruption in the time series. The average level change of the indicator caused by the changeover of the reporting procedure was identified statistically and the time series prior to the changeover was adapted to the level after the changeover. 3) The indicator ‘school leavers without qualification’ was adjusted by impacts of the G8 reforms on the annual numbers of the school leavers. This correction was necessary because the graduating age groups are inflated in those years, in which the first students obtained their school-leaving certificate in the newly introduced G8 system after twelve years. In those years the proportion of the school leavers without qualification is thus lower due to the higher total number of graduates. To subtract these artefacts, the statistical effect of the G8 reforms was estimated by applying regression analyses on the data and was subtracted for the affected federal states in the respective years. 4) When considering the indicator ‘proportion of employees without qualification’, a bimodal distribution became evident, which indicated a significantly lower proportion of employees without qualification in the East German federal states. With regard to content, the indicator should serve as an approximation for the education level of the population. However, historically grown differences between East and West Germany lead to different comparison standards, mainly due to the integration of the GDR population into the employment system of the FRG after the German reunification. The lower proportions of employees without qualification in the East German federal states still originate in stronger obligations of all people to participate in employment and in the education system of the GDR, as a result of which hardly any people did not possess professional qualifications among the population of the GDR [31]. A further reason is the proportion of people with migration history, which was significantly higher among the population of the former FRG, and of which a significantly higher proportion consisted of unskilled and semi-skilled workers. The higher proportion of people without professional qualifications was not only limited to the cohorts of the so-called recruitment agreements of immigrants and their descendants, which immigrated in the 1960 and 1970s, but it pertained in the later immigration movements after 1990 in unified Germany. Meanwhile, in the East German federal states, the immigrant populations since the 1990s saw large shares from Eastern European countries, among which the proportions of people without professional qualifications were lower [32]. The gap between the proportions of the populations without professional qualifications between both parts of the country has thus hardly decreased in the last decades, even if the proportions of people without school-leaving certificate in the East German federal states have been constantly lying above those in the West German federal states. To make corrections for these historically grounded artefacts and demographic differences, the average difference of the corresponding proportions between East and West German federal states was estimated and was added to the regions in the East German federal states, as a result of which a unimodal distribution of the values was obtained. This approach was empirically confirmed as the expected correlations between the indicator with the school leavers without qualification and the proportions of employees with university degree became manifest after this correction was applied.

### 2.3 Index development

The index values were determined in three steps: in a first step, separate principal component analyses were performed for each of the three subdimensions. In a second step, values for the subdimensions were generated with the so-called factor scoring procedure. In a third step, the values for the subdimensions were normalised and were added up to form the GISD score. Just like common factor analysis, the principle component analysis belongs to the dimensionality reduction techniques, which enable to transform several highly correlating indicator variables to one or more factors. In contrast to the common factor model, which most of the factor-analytical methods are based on, the dimensions to be identified are not considered to be causally determining the indicator values in principal component analysis. This also, does not apply for the GISD because regional socioeconomic deprivation is not considered to be the cause for the values of the nine individual indicators, instead the individual indicator values are defining regional socioeconomic deprivation. By means of the principle component analysis the information of the individual indicators is transferred into nine newly generated variables (principal components), so that they successively represent the maximum of information contained in the indicators starting with the first principle component. When the first principal component represents a sufficient amount of variance of the respective indicators, when no further principal component represents variance of substantial size, and when all indicators highly correlate with the first principal component, then the first principal component constitutes a legitimate aggregation of the indicators in one dimension [33]. For the principle component analyses, the indicator data for the GISD were pooled for the years 2001 to 2019. Hence, in addition to the variation between the regional units, the variation over time was also used to determine the correlations between the indicators. Only data as of 2001 was included in the principle component analysis in order to prevent distortions of the weights caused by imputed values. The principle component analyses confirmed a one-dimensional structure for each partial dimension. The factor loading, which can be read as correlation of the indicators with the principle component, reached satisfactory to good values (>0.6) (Table 3).

In the second step, values (scores) for the subdimensions were calculated for each municipality in the data set based on the so-called factor scoring technique. Factor scoring is a mathematical procedure, which combines the indicator values proportionately to the weights identified in the principle component analyses. Concrete values for the sub-dimensions were calculated in this way. Thereby, it was possible to also consider values for the years 1998 to 2000. The factor values of the three subdimensions were subsequently normalised on a yearly basis to range between 0 and 1. The three sub-scores were then added up, so that each subdimension went into the total index with a weight of approximately one third. The GISD scores for the municipalities, which resulted in this way, were subsequently aggregated to higher regional units (GVB, districts and independent cities, spatial planning regions, and the statistical regions according to the official European statistic NUTS-2) based on the weighted population to calculate GISD scores for each regional level. The calculation of the GISD scores by means of population-weighted aggregation ensured that the same indicator weighting was applied to calculate the GISD scores for each spatial level. Based on the district level, values for further spatial references, such as zip code areas, were also generated in this way. The values were normalised at the respective levels on a yearly basis in such a way that the socioeconomic deprivation takes on values between 0 (lowest deprivation) and 1 (highest deprivation) on each spatial level. For the further analyses, the units of the mentioned spatial levels were additionally divided into five groups of twenty percent each for each year (quintiles, fifths) according to the distribution of the index values, whereby the lowest fifth in each case indicates ‘low’, and the highest fifth indicates ‘high’ socioeconomic deprivation.

### 2.4 Analysis strategy

The regional distribution of the index is illustrated below for different administrative spatial levels. Correlations at the district level between regional socioeconomic deprivation and life expectancy as well as cardiovascular mortality and lung cancer incidences are subsequently shown as examples for the application in social epidemiological analyses, in which spatially aggregated health data is linked to the GISD.

To statistically adjust differences in the age structure between regions with a low and high level of deprivation, the cardiovascular mortality and lung cancer incidence was age-standardised using the 2013 European standard population [34]. The age-standardised rates for cardiovascular mortality, classified by means of the ICD-10-GM diagnoses I00–I99 (international statistical classification of the diseases and related health problems, 10th revision, German modification) were calculated from the notified deaths in the official Cause-of-death statistics and data from the population projection from 2003 to 2019. The GISD was used to analyse the socioeconomic inequalities, and the mortality rates for three groups was illustrated: low socioeconomic deprivation (first GISD quintile), average socioeconomic deprivation (second to fourth GISD quintile), and highest socioeconomic deprivation (fifth GISD quintile).

Data from the Centre for Cancer Registry Data from 2012 to 2015 was combined with the district-level GISD to estimate relative risks of lung cancer according to regional socioeconomic deprivation. The analyses were conducted separately for women and men. Age structure effects were controlled in multilevel Poisson regression models with 5-year age groups as first level units and districts as second level units. The varying population size across districts was taken into account by including the logarithmised number of inhabitants as a so-called offset term in the regression models [21]. Women and men from regions with the lowest deprivation level in each case served as reference category.

The calculations relating to the cardiovascular mortality with data from the cause-of-death statistics were performed in the Research Data Centre of the Office for Statistics of Berlin-Brandenburg. The raw data material for the version GISD 2022 v1 introduced here (if freely available), the code for the generation and the generated GISD data can be accessed under the following URL: https://doi.org/10.5281/zenodo.6840304. The generation of the GISD was performed using the statistical package R (version 4.1.3). The correlation analyses were likewise performed using R and Stata SE 17.

## 3. Results

### 3.1 Regional distribution

**Figure 1:**
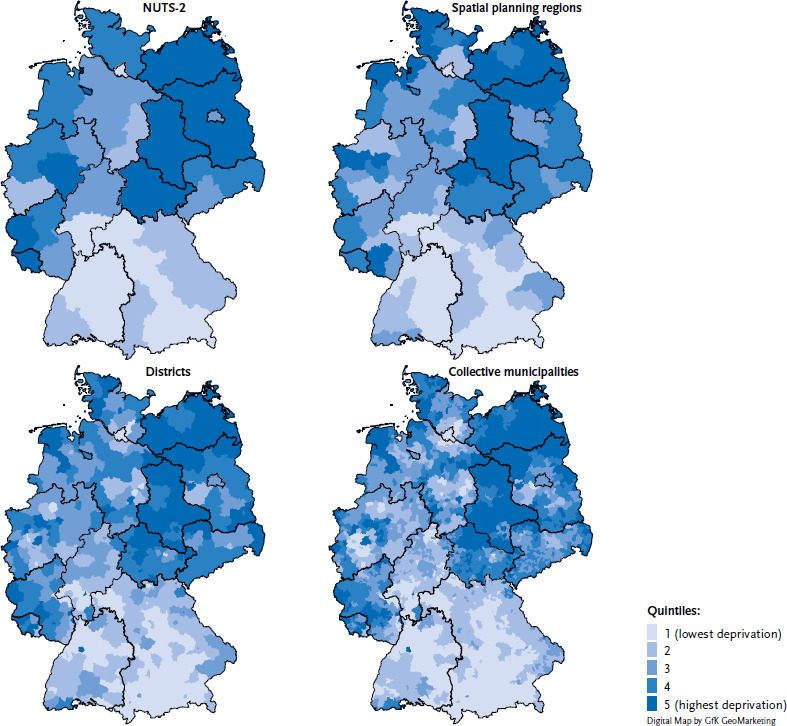
Regional socioeconomic deprivation (in quintiles) at various spatial levels in Germany in 2019 Source: INKAR 2021, Statistics of the Federal Employment Agency, own calculations

The geographical distribution of regional socioeconomic deprivation according to the GISD for the year 2019 shows spatial clusters of regions with high and low socioeconomic deprivation at different levels (Figure 1). Regions with high deprivation are primarily located in the rural areas in the north and northeast of Germany, in particular in Mecklenburg-Vorpommern, Saxony-Anhalt, and in some areas of Berlin-Brandenburg as well as near the coasts of Lower Saxony and Schleswig-Holstein. Further clusters of high socioeconomic deprivation can be found in regions that were affected by economic structural change, for example in the Ruhr area, the region around Aachen, in Rhineland-Palatinate, and in Saarland. Regions with lower levels of deprivation on the other hand, can be found in Bavaria, Baden-Württemberg, in the region around Hamburg, in southern Hesse, and in parts of North Rhine-Westphalia, for instance in Düsseldorf and the Cologne/Bonn region. It is noteworthy that in East Germany, in particularly urban cities deviate from the regionally prevailing pattern of high deprivation. On the other hand, urban districts with high socioeconomic deprivation can be found in the west and south of Germany, where low deprivation is widespread otherwise.

### 3.2 Applications

#### Life expectancy

**Figure 2:**
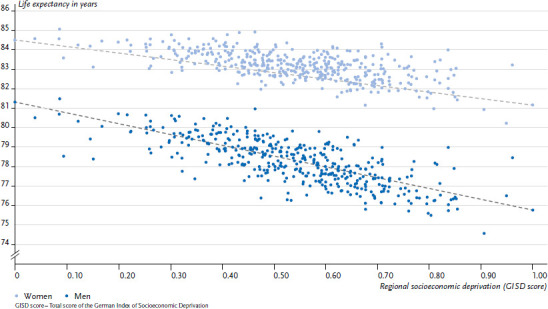
Average life expectancy at birth (2015/2017) at the district level by sex and regional socioeconomic deprivation in 2016 Source: INKAR 2021, Statistics of the Federal Employment Agency, Own calculations

Life expectancy is considered to be the most conclusive indicator of a population’s health status. Between 2015 and 2017, the average life expectancy of a female newborn in Germany was 83.2 years, and 78.4 years for a male newborn [35]. Small-area differences in life expectancy in Germany have also been known for a long time [3, 36–40]. The GISD enables researchers to illustrate to what extent they are associated with the level of regional socioeconomic deprivation. Looking at the average life expectancy at birth of women and men between the years 2015 and 2017 at district level, it is evident that life expectancy decreases with increasing regional deprivation (Figure 2). Particular low levels of life expectancy are found in districts with the highest levels of socioeconomic deprivation. This applies equally for both sexes. Among men, the maximum range between districts with the highest and lowest levels of deprivation are higher than among women. The estimated difference in life expectancy between the least and most deprived district is 6.0 years among men and 3.2 years among women. At the district level, the GISD was able to account for 44.6% of the regional variation in life expectancy among women and 62.0% among men (adjusted R^2^).

#### Cardiovascular mortality

**Figure 3:**
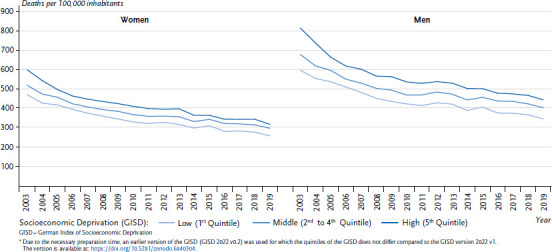
Age-standardised rates of cardiovascular mortality by sex and quintiles of regional socioeconomic deprivation Source: INKAR 2021, Statistics of the Federal Employment Agency, data from the Cause-of-death statistics of the Federal State Offices, Own calculations

In Germany, cardiovascular diseases rank among the most frequent causes of death [41]. Analyses show a clear association of the risk of disease and mortality with socioeconomic status (e.g. [42]). Overall, age-standardised rates of cardiovascular mortality decrease substantially over time, irrespective of sex and GISD category, with mortality significantly higher among males than among females (Figure 3). Individuals living in districts with high socioeconomic deprivation have a higher mortality than individuals in districts with lower socioeconomic deprivation. The regional socioeconomic gradient in cardiovascular mortality persists over the entire observation period. Over time, the decrease of standardised mortality rates was strongest among women and men from districts with the highest socioeconomic deprivation, with drops from 473 (women) and 599 (men) per 100,000 inhabitants to 262 and 350 per 100,000 inhabitants, respectively. The difference corresponds to an absolute decline in the rates by 281 among women and 369 among men (relative decline 47% and 45%, respectively). Among people from districts with middle and low deprivation, the reductions are also substantial. In districts with low deprivation, declines of the rates from 211 among women and 249 among men have been observed, in districts with middle deprivation from 220 among women and 273 among men (relative decline for low deprivation at 45% and 42%, respectively, for middle deprivation at 42% and 40%, respectively).

#### Lung cancer incidence

**Figure 4:**
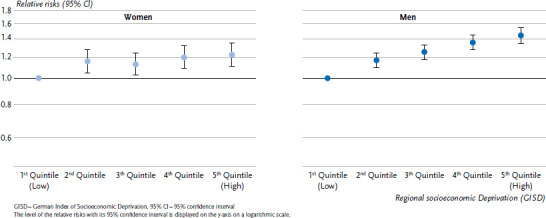
Age-standardised relative risks (RR) of lung cancer incidence by sex and quintiles of regional socioeconomic deprivation for the time period 2012 to 2015 Source: INKAR 2021, Statistics of the Federal Employment Agency, Data from the Centre for Cancer Registry Data at the RKI, Own calculations

Due to its high lethality, lung cancer is likewise one of the most frequent causes of death and is also responsible for a high proportion of years of life lost [41]. Studies have reported substantial socioeconomic differences in lung cancer survival and mortality [21, 43, 44]. Stratified according to GISD quintiles, clear regional socioeconomic inequalities can be found in the risks of lung cancer incidence among men (Figure 4). With increasing deprivation quintile, the relative risk of lung cancer incidence increases continuously compared to the lowest deprivation quintile (reference group). In the highest deprivation quintile, the relative risk is 46% higher than in the lowest deprivation quintile. Among women, the socioeconomic inequality is less pronounced. Women from the highest deprivation quintile have a 23 % increased incidence risk compared to women in the reference group (Figure 4).

## 4. Discussion

### 4.1 Summary

Five years after the initial publication of the German Index of Socioeconomic Deprivation (GISD), the present article introduces its current revision and recent optimisations. The GISD is constructed as an index for measuring the extent of relative regional socioeconomic deprivation in German regions, which merges the dimensions education, employment, and income with equal weights. The GISD scores represent the relative position of a region compared to the regions with the best and the worst socioeconomic situation. The GISD is generated at the municipality level and is provided for further spatial references based on population-weighted aggregations. The approach for the generation of the GISD follows the standards of the national and international literature [45]. Indices for New Zealand [46], Canada [47], France [48], Denmark [49], and China [50] also use principle component analyses to weight indicators within different dimensions of regional socioeconomic deprivation. Beyond the update of the GISD for the period from 1998 to 2019, one indicator addition and some harmonisations of the previously used indicators were made in the current revision. In the future, the GISD is to be updated at regular intervals. The GISD can be freely accessed using the following link: https://doi.org/10.5281/zenodo.6840304.

The association between life expectancy and regional socioeconomic deprivation was shown in the exemplary analyses in this article. Socioeconomic differences in life expectancy and mortality are particularly comprehensible and can be considered as an endpoint where socially induced inequalities of health chances culminate in. Cardiovascular diseases and lung cancer rank among the most frequent causes of death and have the largest proportion of years of life lost for both sexes [41]. The social gradients presented for lung cancer incidences and deaths due to cardiovascular diseases suggest that regional differences in risks of disease and death can be attributed to socioeconomic differences to a considerable degree. It can be assumed that the sex differences observed in the examples relating to life expectancy, cardiovascular mortality, and cancer incidence, can be attributed to differences in the sex-related health and risk behaviour for both diseases and life expectancy in general. Compared to women, men do not only show higher prevalence rates for cardiovascular diseases and for lung cancer, but also for the primary risk factors of these diseases (e.g. excess weight, smoking) [51–53]. As a whole, the illustrated findings reflect the results of the national and international literature. A worse health situation and a more unfavourable health behaviour can thus be found in regions with higher socioeconomic deprivation [10, 54–56]. The pattern of higher mortality and lower life expectancy with increasing socioeconomic deprivation of a region was also determined in other highly developed countries [57–59].

Socio-spatial aggregate data analyses with the GISD or related measures of spatially distributed socioeconomic situations are useful in all situations in which individual data on the socioeconomic position are missing and where population-representative samples are not large enough to analyse health chances and disease risks stratified by socioeconomic categories, i.e. for health outcomes with low prevalence. By using the GISD, regional socioeconomic differences in the nationwide occurrence of cancer in Germany were documented for the most important cancer types for the first time [21]. It could be shown that type and extent of the socioeconomic differences vary depending on the type of cancer and between women and men. In addition to incidence rates, oncological research also analysed survival time rates with regard to social differences. The results of such studies generate insights for care and prevention research and can help to improve survival chances and to reduce disease burdens as a whole.

Official notification data for various infectious diseases [23–25] and ambulatory claims data [26] represent further data sources, for which socioeconomic inequalities could be analysed by linking them to regional deprivation measures. During the Coronavirus pandemic in 2020 and 2021, the use of the GISD enabled the observation of social inequalities in infection and mortality rates on a weekly basis over the course of the pandemic. For the different infection waves, the analyses revealed a recurring pattern, according to which the age-standardised incidence and death rates initially appeared to be higher in regions with a lower level of deprivation, while this pattern reversed in the later course of the infection waves to the disadvantage of regions with higher deprivation [23, 60, 61]. A social gradient in favour of regions with a higher deprivation was also determined for measles incidence in Germany [24]. Further data sources offering potentials for such data analyses at the aggregate level include, for example, the Diagnosis-Related Group Statistics (DRG Statistics), the data of the Cause-of-death statistics (TUS), which can be obtained from the statistical offices of the German states, and indicators provided by the RKI relating to the years of life lost due to sickness and death (disease burden) (https://www.daly.rki.de/) [41, 62].

Insights about the accordance of regional socioeconomic deprivation levels with individual data can be obtained from multilevel analyses. A significant part of the socio-spatial correlations between deprivation and health outcomes is generally reduced when individual socioeconomic indicators are controlled for. However, the correlation between regional deprivation and health remained significant in the respective studies [10, 11, 27]. These findings confirm the assumption that contextual influences beyond the individual socioeconomic status affect health. Factors are assumed here, which directly correlate with the wealth of the residential area and which depend on economic and political conditions [9]. In addition to environmental conditions, the availability of health care and preventive services, the literature also refers to the dissemination of health-related standards and values as well as social cohesion [55, 56, 63].

### 4.2 Limitations

The chosen approach for generating the GISD offers several advantages and expanded options for health reporting and (social) epidemiology in Germany, but also has restrictions and limitations. For instance, the granularity of the used data is highly dependent on the population size of the municipality. Within large city districts, a differentiation between neighbourhoods is not possible because the lowest regional unit in these cases coincides with the respective district or even with the federal state. For further resolution, it would be necessary to find and harmonise matching data at the smaller district or neighbourhood level. A further limitation relates to the lack of indicators at the GVB level. Variation below the district level for the education dimension could not be represented in the GISD at all. The potential strength of correlations with the GISD at the municipality level is thus substantively limited. The spatial distribution at the GVB level provides an impression about the fact that the GISD nonetheless detects substantial variation within districts (Figure 1).

Due to the limited data situation, it must be assumed that the process-produced data used for the GISD are inferior to the census data used in many international indices for measuring socioeconomic deprivation because the distributions of socioeconomic features in the regions with the individual data from the census are measured directly and thus more accurately while the range of process-produced data from the official statistics for the small-area level of municipalities remains restricted. In particular the income dimension would benefit from indicators which better represent the lower income bracket and poverty. Due to the irregular availability of data from census surveys in Germany, the used data remains without alternative for the time being. The data from the census conducted in 2022, however, will permit an evaluation of the GISD based on microdata. A further distinctive feature relates to the restriction of the GISD to socioeconomic indicators. In addition to socioeconomic indicators, further health-relevant factors, such as environmental burden, crime rate, and political engagement are considered in indices of multiple deprivation. One example for such an index of multiple deprivation is the German Index of Multiple Deprivation (GIMD) [64]. Indices of multiple deprivation perform better in detecting regional differences in the need for care than purely socioeconomic deprivation indices. However, there is a risk for the (social) epidemiological research that the causes of good health and health risks are conceptually mixed with the consequences of poor health and disease [14]. This applies in particular when, for example, the life expectancy of the regional population or other health characteristics, such as the proportion of households with chronically ill people, are included in such an index, and the index is then used to explain regional differences in the life expectancy [14]. A purely socioeconomic index of deprivation without a health component thus provides analytical advantages, even though socioeconomic disadvantages could generally also result from poor states of health. In summary, a concept was pursued for the GISD, which is based on theories relating to socioeconomically determined health inequalities and which allows for an analogy to the individual socioeconomic status with its partial dimensions education, employment, and income.

The so-called ecological fallacy is the core issue of the correlation analyses at regional level. As a result, conclusions from the correlational analyses are limited to the identification of disadvantaged regions, which prevents the drawing of conclusions to the causal effect of individual disadvantage [15, 18, 45]. This problem cannot be solved conclusively. After all, the use of the index at the level of the municipalities increases the socioeconomic homogeneity compared to the district level, and there is evidence that health inequalities are underestimated more strongly, the more coarsely the spatial level is resolved [45, 65]. The most important goals of analyses with the GISD are to directly derive research hypotheses for the individual level from the findings of regional aggregate analyses, to describe local and regional potentials for prevention which are related to the social structure, as well as to supplement the social epidemiological research where data availability is restricted. In international comparison, Germany has a significantly limited data situation for analyses relating to social inequalities in mortality and life expectancy. While data from census-based mortality follow-ups or population-based registers are available in many countries, which include information relating to the mortality and socioeconomic situation of individuals [66, 67], this is not the case in Germany. Because of this data gap, social epidemiological research on these important health outcomes is limited. Establishing a national mortality register with linking options to census or social insurance data at the individual level could remedy this gap. Analyses, such as those represented in this article, currently remain the only option for describing and documenting social inequalities in (cause-specific) mortality and life expectancy at a nationwide scale in Germany.

### 4.3 Conclusion

The wide range of analyses, which were performed using the GISD and other regional deprivation indices, and which will become possible in the future, confirm that the GISD represents a valuable addition to the social epidemiological research and health reporting in Germany. They also document the potentials for gaining knowledge with high public health relevance [10]. A particular added value of socio-spatial analyses using the GISD lies in uncovering regional contextual impacts on health, which are considered only marginally in social epidemiology up until now. The continuation of the GISD’s free public release and further efforts to its improvement remain important goals. The GISD will furthermore be used for nationwide surveillance systems, which are currently prepared at the RKI [68], aiming at a continuous description and monitoring of social differences in non-communicable diseases (NCD) over time. In the context of infectious disease epidemiology, the GISD enables to instantaneously document socioeconomic inequalities in dynamic outbreaks. Results of the analyses with regional deprivation indices can be used to inform health-policy strategies and measures for promoting health and prevention, in particular in socioeconomically disadvantaged regions.

## Key statement

- The GISD illustrates regional socioeconomic inequalities on various spatial levels over the course of time.
- The index is based on nine indicators which reflect the three subdimensions of deprivation: education, employment, and income.
- Regions with high socioeconomic deprivation tend to be located in the north and northeast of Germany, but are also found in the Saarland, Rhineland-Palatinate, and North Rhine-Westphalia.
- A lower life expectancy as well as a higher cardiovascular mortality and lung cancer incidences are found in regions with higher regional socioeconomic deprivation.
- The index can be used for the continuous analysis and reporting of health-related inequalities and makes it possible to tap into new primary and secondary data sources.

## Figures and Tables

**Table 1:** Spatial levels in Germany and average population figures Source: INKAR 2021, individual calculation

Level	Number	Average population	Minimum	Maximum
Municipalities	10,799	7,701	10	3,669,491
Municipalities and Collective municipalities (GVB)	4,411	18,854	324	3,669,491
Districts and independent cities (districts)	401	207,398	34,193	3,669,491
Spatial planning regions (ROR)	96	866,320	194,363	3,669,491
NUTS-2	38	2,188,598	533,113	5,207,457

NUTS-2 = Nomenclature des unités territoriales statistiques: EU statistical regions, level 2, basic regions. Corresponds to the administrative districts or statistical regions of the federal states of Germany. Territorial and population status as of 31 December 2019

GVB = single municipalities and collectives of smaller municipalities (Gemeindeverbände)

**Table 2:** Indicators of the socioeconomic deprivation Source: INKAR 2021, Statistics of the Federal Employment Agency [29]

Dimension	Characteristic	Indicator	Source	Availability
Education	Employees with university degree	Proportion of employees subject to social insurance contributions at place of residence with university degree as share of total employees subject to social insurance contributions at place of residence in %	Statistics of the Federal Employment Agency	Districts for the years 2001–2011 and 2013–2019^*^
Education	Employees without qualification	Proportion of employees subject to social insurance contributions at place of residence without professional qualification as share of total employees subject to social insurance contributions at place of residence in %	Statistics of the Federal Employment Agency	Districts for the years 2001–2011 and 2013–2019^*^
Education	School leavers without qualification	Proportion of school leavers without lower secondary school leavers certificate out of all school leavers in %	Statistics on the schools of general education of the federal and state governments	Districts for the years 1998–2019
Employment	Unemployment rate	Proportion of unemployed as share of inhabitants of working age	Statistics of the Federal Employment Agency	GVB for the years 1998–2019^**^
Employment	Employment rate	Employees subject to social insurance contributions at place of residence per 100 inhabitants of working age	Statistics of the Federal Employment Agency	GVB for the years 1998–2019^**^
Employment	Gross wage and salary	Monthly gross earnings of employees in EUR	National Accounts of the Federal States	Districts for the years 2000–2019
Income	Net household income	Average household income in € per inhabitant	National Accounts of the Federal States	Districts for the years 2000–2019
Income	Debtor quota	Private debtors per 100 inhabitants aged 18 and above in %	Statistics from creditreform e.V. associations in Germany	Districts for the years 2004–2019
Income	Tax revenue	Income tax in € per inhabitant	Comparison of federal and federal state taxation on real estate and working assets	GVB for the years 1998–2019

^*^ Data source for the proportions of employees subject to social insurance contributions without qualification and with university degree is the Statistics of the Federal Employment Agency [29]. Data for the years 2013 to 2019 is freely available there. Data for the years 2001 to 2011 was obtained directly via the Statistics of the Federal Employment Agency.

^**^ For unemployment and employment rates, values for the GVB level were only available as of 2001. For the years 1998 to 2001, the values of the district level were assigned to the GVB.

GVB = comprises large municipalities and collectives of smaller municipalities (Gemeindeverbände)

**Table 3:** Weighting of the indicators for the German Index of Socioeconomic Deprivation (GISD) Source: INKAR 2021, Statistics of the Federal Employment Agency, Own calculations

Dimension	Variable	Factor loading	Share in the dimension	Share GISD
Education 33.3%	Employees with university degree	-0.732	34.1	11.4
	Employees without qualification	0.771	37.8	12.6
	School leavers without qualification (adj.)	0.663	28.0	9.3
Employment 33.3%	Employment rate	-0.640	23.1	7.7
	Unemployment rate	0.841	39.9	13.3
	Gross earnings (log.)	-0.810	37.0	12.3
Income 33.3%	Income tax (log.)	-0.914	40.7	13,6
	Household income (log.)	-0.921	41.3	13.8
	Debtor quota	0.608	18.0	6.0

Description of the factor loadings on the first principle component for the subdimensions. Eigenvalues of the first components: η_Education_=1.6; η_Employment_=1.8; η_Income_=2.0. The eigenvalues of the second and third components are below 0.8 in each case. The correlations between the scores of the subdimensions for the year 2019: r_Employment|Income_=0.66; r_Employment|Education_=0.52; r_Income|Education_=0.69. log. = logarithmised, adj. = adjusted
